# Lactoferrin Has a Therapeutic Effect *via* HIF Inhibition in a Murine Model of Choroidal Neovascularization

**DOI:** 10.3389/fphar.2020.00174

**Published:** 2020-02-28

**Authors:** Mari Ibuki, Chiho Shoda, Yukihiro Miwa, Ayako Ishida, Kazuo Tsubota, Toshihide Kurihara

**Affiliations:** ^1^ Laboratory of Photobiology, Keio University School of Medicine, Tokyo, Japan; ^2^ Department of Ophthalmology, Keio University School of Medicine, Tokyo, Japan; ^3^ Department of Ophthalmology, Nihon University, Tokyo, Japan; ^4^ Tsubota Laboratory, Inc., Tokyo, Japan

**Keywords:** lactoferrin, age-related macular degeneration, laser-induced choroidal neovascularization, hypoxia-inducible factor, retina, retinal pigment epithelium, choroid

## Abstract

**Background:**

Lactoferrin, a type of glycoprotein, is contained in exocrine fluids such as tears, breast milk, sweat, and saliva, and is known to have anti-microbial, antioxidant, and anti-cancer effects. In the ophthalmological field, topical administration of lactoferrin has been reported to have a therapeutic effect in a murine dry eye model. Hypoxia-inducible factor (HIF) regulates various gene expressions under hypoxia, including vascular endothelial growth factor (VEGF), and is considered as an alternative target for neovascular ocular diseases such as age-related macular degeneration (AMD). We previously screened natural products and identified lactoferrin as a novel HIF inhibitor. In this study, we confirmed that lactoferrin has an HIF inhibitory effect and a therapeutic effect in a murine model of neovascular AMD.

**Methods:**

HIF inhibitory effects of lactoferrin were evaluated using a luciferase assay and western blotting *in vitro*. The quantified volume of choroidal neovascularization (CNV) induced by laser irradiation was compared with oral lactoferrin administration or conditional tissue specific *Hif1a* knockout mice.

**Results:**

Lactoferrin administration showed a significant HIF inhibitory effect in the retinal neuronal cells. Oral administration of lactoferrin or conditional *Hif1a* gene deletion significantly reduced CNV volume compared to controls.

**Conclusions:**

Lactoferrin has a therapeutic effect in a laser CNV model by suppressing the retinal HIF activity.

## Introduction

Lactoferrin, a type of glycoprotein, is contained in exocrine fluids such as nasal exudate, bronchial mucus, breast milk, tears, sweat, and saliva (Iigo et al., 2009). The concentration of lactoferrin varies in different human body fluids. Milk is the most abundant source of lactoferrin, with human colostrum containing up to 7 g/l (Masson and Heremans, 1971). The concentration in tears is 2 mg/ml, whereas that in blood is normally only 1 μg/ml, although it can rise to 200 μg/ml in an inflammatory situation (Masson and Heremans, 1971). It is reported that lactoferrin is responsible for several anti-infective, immunological, and gastrointestinal actions in neonates, infants, and young children (Manzoni et al., 2018). Lactoferrin is also known to have several biological functions, including antioxidant, anti-microbial, and anti-cancer effects (Kanwar et al., 2015). It is reported that oral administration of bovine lactoferrin inhibits carcinogenesis in the colon and other organs in rats, and lung metastasis in mice (Iigo et al., 2009). In the ophthalmological field, lactoferrin eye drops have been reported to have a therapeutic effect in a murine dry eye model by suppressing oxidative stress (Higuchi et al., 2012; Higuchi et al., 2016).

Age-related macular degeneration (AMD) is a leading cause of blindness globally. It is roughly classified into two types; atrophic type (dry AMD) and neovascular type (wet AMD). Wet AMD is characterized by neovascularization, and vascular endothelial growth factor (VEGF) is known as a major contributor to the pathogenesis. While treatment for wet AMD with anti-VEGF drugs is established and widely clinically performed, there exist some concerns of adverse effects with long-term administration, such as chorioretinal atrophy (Grunwald et al., 2014; Maguire et al., 2016).

Hypoxia-inducible factors (HIFs) are key molecules regulating various gene expressions, including VEGF, which are required for cell survival under hypoxia. HIFs are transcriptional factors that are stabilized and activated under hypoxic conditions (Wang and Semenza, 1995). Under normoxic conditions, α-subunits of HIFs are hydroxylated by prolyl hydroxylase, ubiquitinated by von Hippel- Lindau (VHL) protein recognition, and degraded in the proteasome. Under hypoxic conditions, the activity of HIF-α prolyl hydroxylase decreases and HIF-αs are stabilized (Kaelin and Ratcliffe, 2008). We have previously revealed physiological and pathological roles of HIFs in the developmental and adult retina (Kurihara et al., 2010; Kurihara et al., 2011; Kurihara et al., 2016). Retinal pigment epithelium (RPE) cells are important to maintain homeostasis in the retina, and contribute to the pathogenesis of AMD (de Jong, 2006). RPE-specific conditional *Vegf* knockout mice show choriocapillaris loss, RPE and photoreceptor cell degeneration, and subretinal deposit accumulation resembling human AMD phenotypes (Kurihara et al., 2012; Kurihara et al., 2016). In contrast, RPE-specific *Hif* knockout mice show no pathological phenotypes morphologically and functionally, although both *Vegf* and *Hif* RPE-specific knockout mice have a significant and similar reduction of laser-induced choroidal neovascularization (CNV) mimicking wet AMD (Kurihara et al., 2012).

To identify dietary factors inhibiting HIF and examine the functions against ocular diseases, we have screened natural products and reported their therapeutic effects in animal models of retinal disorders (Kunimi et al., 2019a; Kunimi et al., 2019b; Miwa et al., 2019). We further screened natural products from another library and revealed that administration of *Garcinia cambogia* extract and its main ingredient hydroxycitric have HIF inhibitory effects, showing significant therapeutic effects in a murine laser-induced CNV model (Ibuki et al., 2019). Furthermore, another group also revealed that *in vivo* genome editing targeting HIF could suppress laser-CNV formation in mice, indicating that HIF inactivation in the retina may be a promising approach to treat the neovascular type of AMD (Kim et al., 2017).

From the screening test, we have identified lactoferrin can be a novel candidate to inhibit HIF. In this study, we confirmed that lactoferrin has an HIF inhibitory effect, especially in retinal neuronal cells. In addition, we revealed a pathological contribution of HIF, not only in RPE cells but also neuronal cells in sensory retina, by utilizing murine models of conditional gene deletion.

## Materials and Methods

### Animals

We performed all procedures in accordance with the National Institute of Health (NIH) guidelines for work with laboratory animals, the ARVO Animal Statement for the Use of Animals in Ophthalmic and Vision Research, and the Animal Research: Reporting *in vivo* Experiments (ARRIVE) guidelines. Our all animal procedures were approved by the Institutional Animal Care and Use Committee at Keio University. Wild-type C57BL6/J mice (CLEA Japan, Tokyo, Japan) and other transgenic mice were raised in an air-conditioned room maintained at 23 ± 3°C under a 12 h dark/light cycle, with free access to food and water.

Transgenic mice expressing Cre recombinase under Best1 [Best1-Cre mice, (Iacovelli et al., 2011)] or Chx10 promoter [Chx10-Cre mice, (Muranishi et al., 2011)] were mated with *Hif1a* ^flox/flox^ mice (Ryan et al., 1998) to obtain RPE or sensory retina specific *Hif1a* knockout mice, respectively. *Hif1a* ^flox/flox^ mice without the Cre transgene were used as the control. The genetic background of all transgenic mice used in this study was C57BL6/J.

### Luciferase Assay

We performed a luciferase assay as previously described (Ibuki et al., 2019). The luciferase assay was performed using the murine cone photoreceptor cell line (661W) and the human RPE cell line (ARPE19). HIF-αs were induced by 200 μM CoCl_2_. Lactoferrin (FUJIFILM Wako Pure Chemical) was dissolved in MQ so that its concentration was 1 mg/mL, and was added into the growth medium at the same time as CoCl_2_. After the administration, cells were incubated for 24 h and the luciferase expression was quantified. We used a total of 100 μM of topotecan (Cayman Chemical, Ann Arbor, MI, USA) as a positive control for an HIF inhibitor, and a medium without CoCl_2_ and lactoferrin as a vehicle control.

### Laser-Induced CNV

The laser irradiation was performed as previously described (Ibuki et al., 2019). We dilated the eyes of the mice and anesthetized them. We placed five laser spots (532 nm argon laser, 200 mW, 100 ms, 75 mm). We used the air bubble as an index of Bruch’s membrane disruption by laser irradiation and excluded laser spots without an occurrence of the air bubble from the data analysis. We also excluded laser spots with an occurrence of hemorrhage because those spots may vary in the development of CNV.

### CNV Volume Measurement

We measured CNV volume as previously described (Ibuki et al., 2019). On the 7th day after the irradiation, we sacrificed the mice, and enucleated the eyeballs. The RPE-choroid-sclera complex was flat-mounted and stained with isolectin B4. We observed CNV with a laser microscope, generated three-dimensional images of the CNV, and measured the volume.

### Administration of Lactoferrin to Mice

Lactoferrin was dissolved in PBS to a concentration of 1,600 mg/kg, and was administered to 3-week-old male mice 6 days/week for a total of 5 weeks. The control group was administered PBS. The mice were irradiated with a laser 4 weeks after the beginning of the administration.

### Real-Time PCR

We performed real-time PCR as previously described (Ibuki et al., 2019). We extracted RNA from the ARPE19 cell line and the 661W cell lines. We calculated the relative amplification of the cDNA fragments using the 2-ΔΔCt method. Real-time PCR primer sequences were as follows: human *Hif1a* forward: TTCACCTGAGCCTAATAGTCC, human *Hif1a* reverse: CAAGTCTAAATCTGTGTCCTG; human *Vegfa* forward: TCTACCTCCACCATGCCAAGT, human *Vegfa* reverse: GATGATTCTGCCCTCCTCCTT; *human Glut1* forward: CGGGCCAAGAGTGTGCTAAA, human *Glut1* reverse: TGACGATACCGGAGCCAATG; human *Pdk1* forward: ACAAGGAGAGCTTCGGGGTGGATC, human *Pdk1* reverse: CCACGTCGCAGTTTGGATTTATGC; human *Bnip3* forward: GGACAGAGTAGTTCCAGAGGCAGTTC, human *Bnip3* reverse: GGTGTGCATTTCCACATCAAACAT; human *Gapdh* forward: TCCCTGAGCTGAACGGGAAG, human *Gapdh* reverse: GGAGGAGTGGGTGTCGCTGT; mouse *Hif1a* forward: GGTTCCAGCAGACCCAGTTA, mouse *Hif1a* reverse: AGGCTCCTTGGATGAGCTTT; mouse *Vegfa* forward: CCCTCTTAAATCGTGCCACC, mouse *Vegfa* reverse: CCTGTCCCTCTCTCTGTTCG; mouse *Glut1* forward: CAGTTCGGCTATAACACTGGTG, mouse *Glut1* reverse: GCCCCCGACAGAGAAGATG; mouse *Pdk1* forward: GGCGGCTTTGTGATTTGTAT, mouse *Pdk1* reverse: ACCTGAATCGGGGGATAAAC; mouse *Bnip3* forward: GCTCCCAGACACCACAAGAT, mouse *Bnip3* reverse: TGAGAGTAGCTGTGCGCTTC; mouse *Gapdh* forward: AGGAGCGAGACCCCACTAAC, and mouse *Gapdh* reverse: GATGACCCTTTTGGCTCCAC.

### Western Blot

We performed Western Blot as previously described (Ibuki et al., 2019).

For *in vitro* experiments, we added 200 μM CoCl_2_ and 1 mg/mL lactoferrin to the ARPE19 cell line and 661W cell line. Six hours after the administration, we extracted protein from the cells and adjusted the protein concentration to 75 μg/30 μL.

For the *in vivo* experiments, we sacrificed the mice, and enucleated the eyes on the 3rd day after the laser irradiation. Six ocular samples from three mice were pooled per group. We adjusted the protein concentration to 55 μg/42 μL.

We incubated the membranes with rabbit monoclonal antibodies against HIF-1α, or mouse monoclonal antibodies against β-actin. We washed incubated the membranes with a horseradish peroxidase (HRP)-labeled secondary antibody for HIF-1α, or with a HRP-labeled secondary antibody for β-actin.

### Statistics

We used a two-tail Student’s *t*-test for the comparison of two groups. To compare multiple groups, we used a one-way analysis of variance (ANOVA) followed by Tukey’s post hoc test. Probability values less than 0.05 was considered as being statistically significant. We expressed all results as the mean ± standard deviation.

## Results

### HIF Activation Was Suppressed by Lactoferrin Administration in a Luciferase Assay

**Figure 1:**
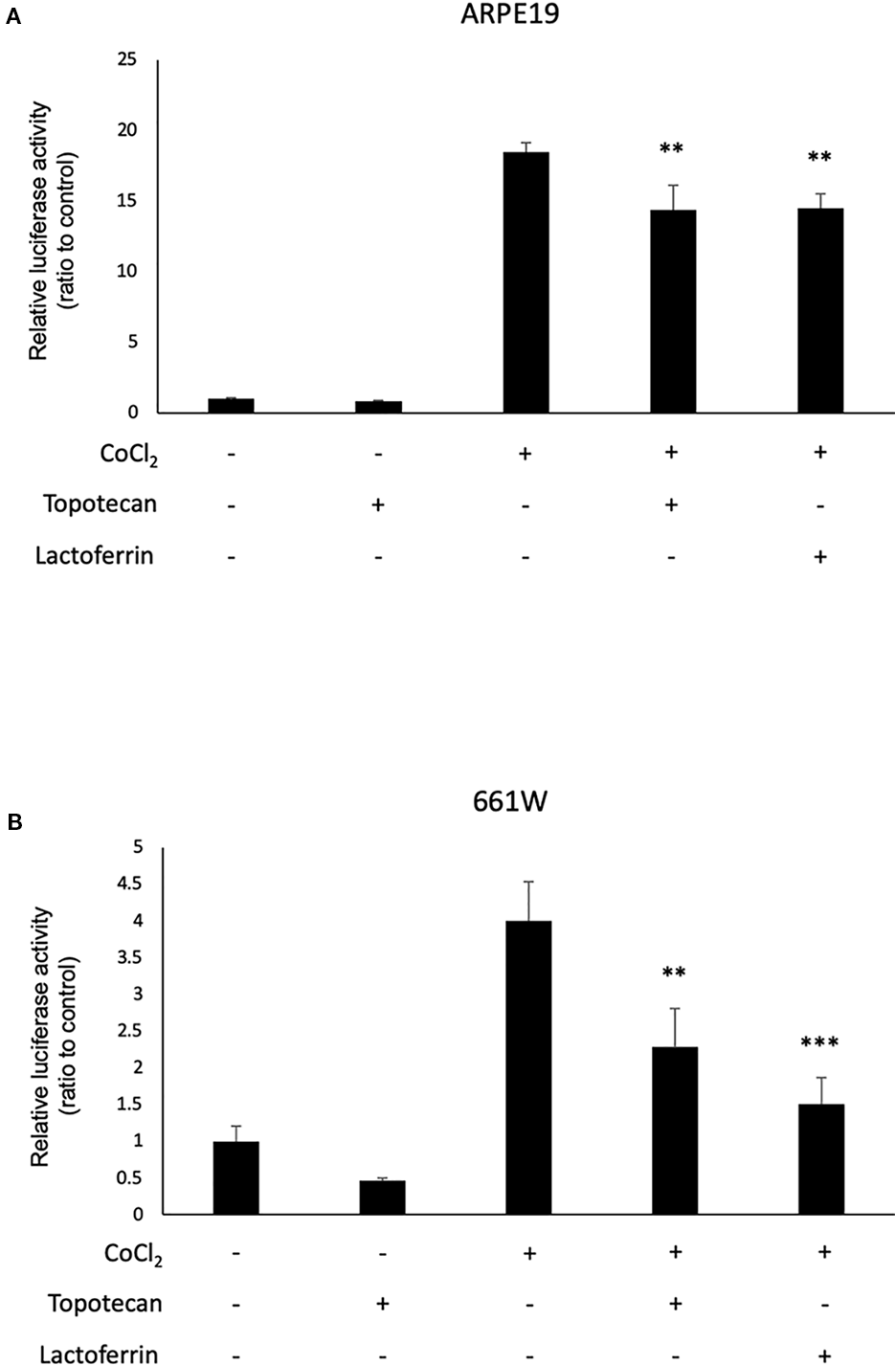
Lactoferrin suppressed hypoxia-inducible factors (HIF) activation in a luciferase assay. **(A)** Administration of lactoferrin significantly suppressed CoCl_2_-induced HIF activation in ARPE19 cells. **(B)** Administration of lactoferrin significantly suppressed CoCl_2_-induced HIF activation in 661w cells. **p < 0.01, ***p < 0.001, compared with CoCl_2_ without topotecan and lactoferrin, n = 3.

We used 661W and ARPE19 to evaluate HIF activity with a luciferase assay. CoCl_2_ was added to activate HIF signaling. Topotecan was used as a positive control of the HIF inhibitor. Lactoferrin showed an HIF inhibitory effect compared with the control group in ARPE19 cells (**Figure 1A**) and 661W cells (**Figure 1B**). Luciferase activity may be affected by magnesium concentration. To examine whether the chelate activity of lactoferrin changes magnesium concentration in the medium, we measured magnesium concentration in the medium with or without lactoferrin and there was no significant change observed (**Figure S4**).

### Administration of Lactoferrin Downregulated *Hif1a* and Its Downstream Genes in 661W Cone Photoreceptor Cells

**Figure 2:**
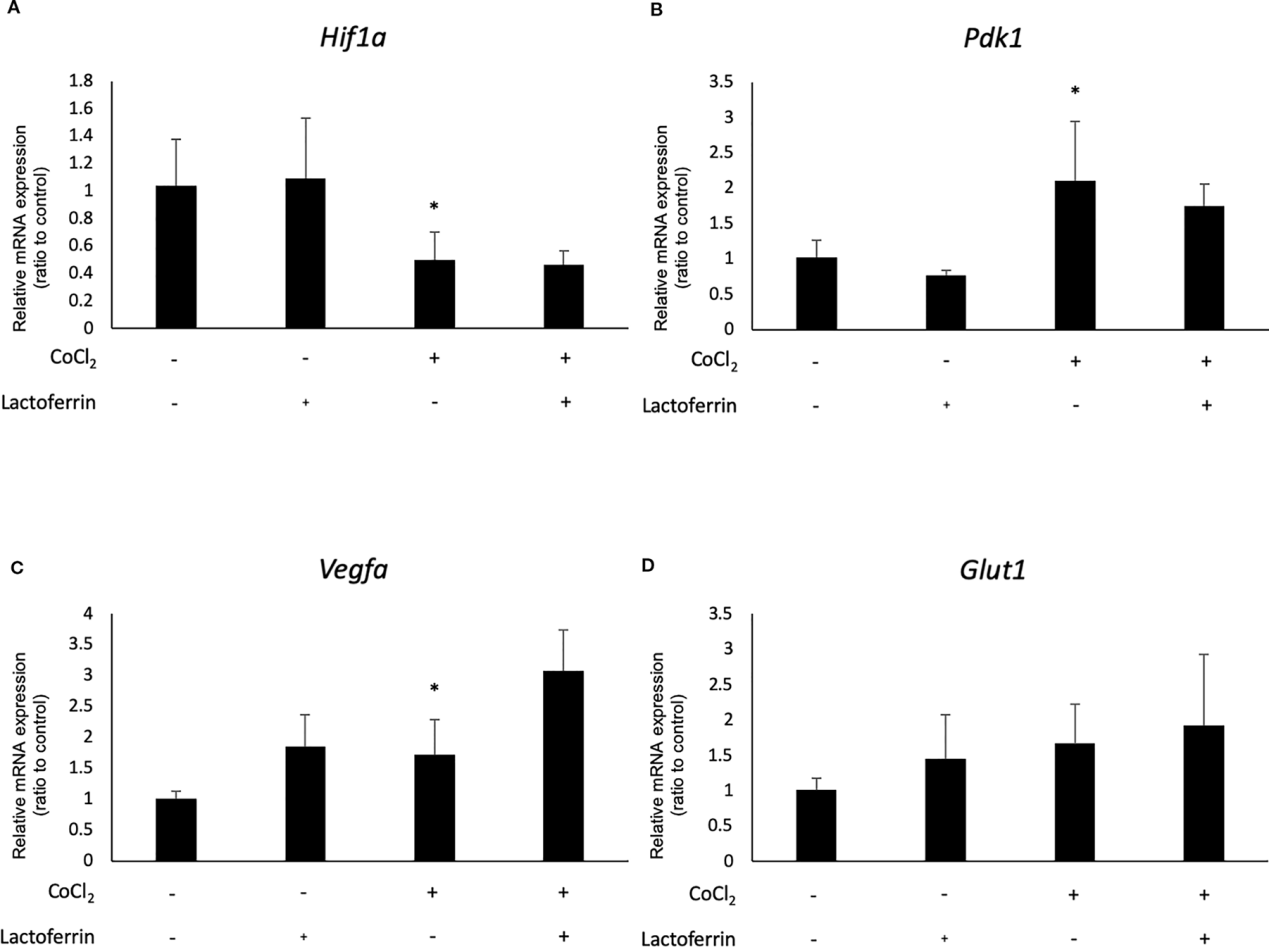
*Hif1a* and the downstream genes were not affected by lactoferrin administration in ARPR19 cells. **(A)** *Hif1a* was downregulated by CoCl_2_ administration in APRE19 cells. The downstream genes of HIFs, including **(B)** *Pdk1*, **(C)** *Vegfa*, and **(D)** *Glut1* were upregulated by the administration of CoCl_2_, but not changed by lactoferrin administration in ARPE19 cells. *p < 0.05, compared with the control, n = 4–6.

**Figure 3:**
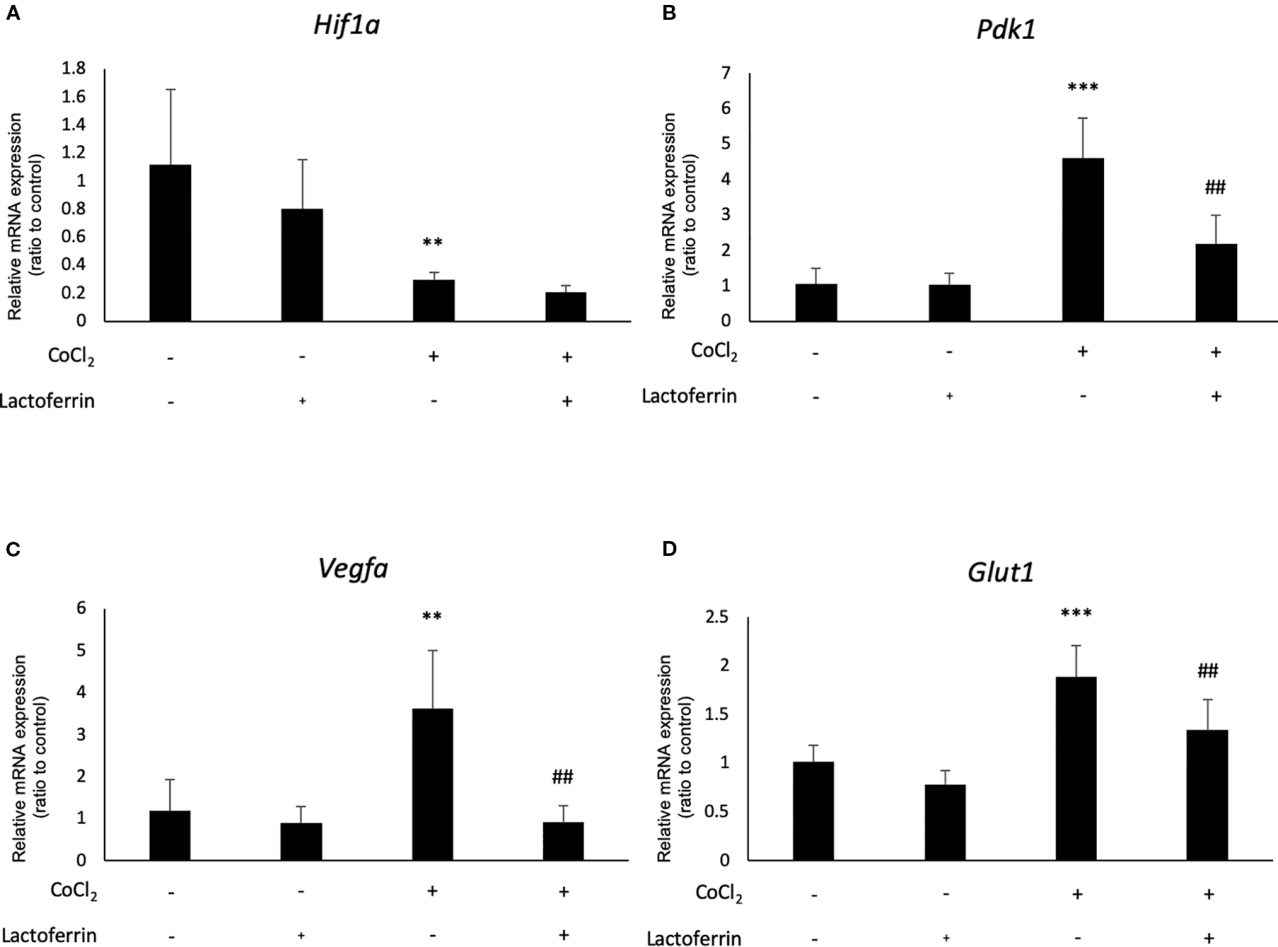
*Hif1a* and the downstream genes were affected by lactoferrin administration in 661W cells. **(A)** *Hif1a* was downregulated by CoCl_2_ administration in 661W cells. The downstream genes of HIFs, including **(B)** *Pdk1*, **(C)** *Vegfa*, and **(D)** *Glut1* were upregulated significantly by the administration of CoCl_2_ and **(B)** *Pdk1*, **(C)** *Vegfa*, and **(D)** *Glut1* were suppressed significantly by lactoferrin administration in 661W cells. **p <0.01, ***p <0.001, compared with the control. ^##^ *p <*0.01, compared with CoCl_2_ without lactoferrin, n = 3–8.

**Figure 4:**
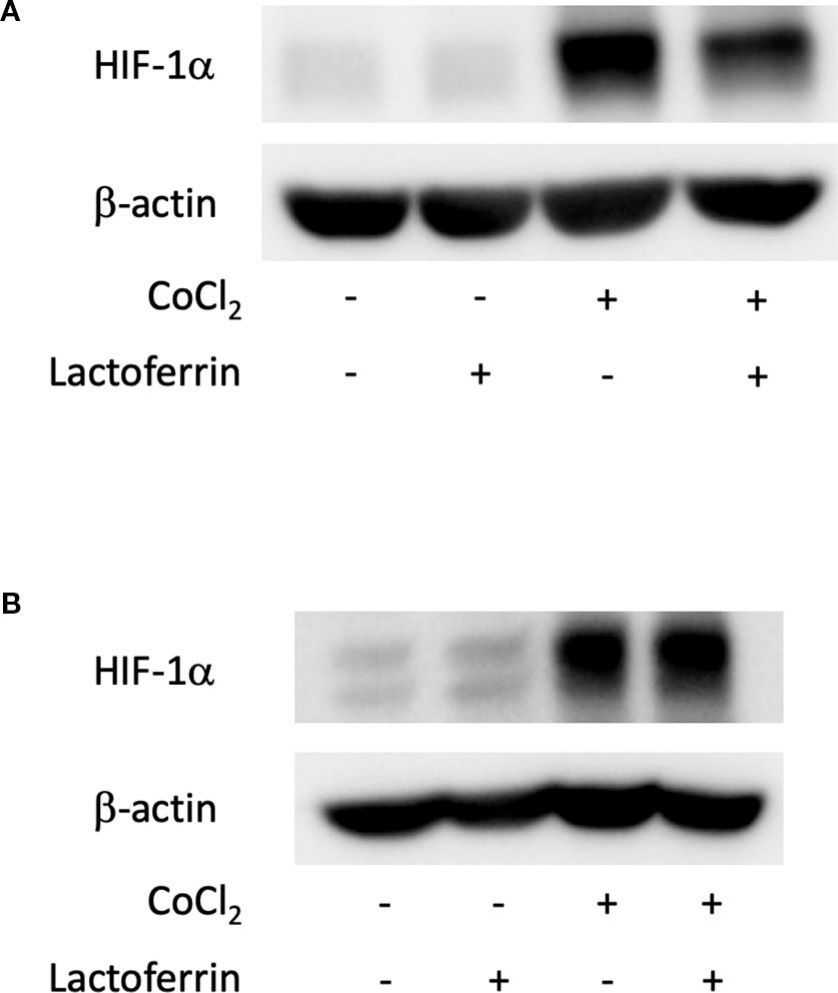
HIF-1α protein expression was not changed by lactoferrin administration in ARPE19 cells and 661W cells. **(A)** Western blot for HIF-1α and β -actin in ARPE19 cells. **(B)** Western blot for HIF-1α and β -actin in 661W cells.

We examined how lactoferrin affects mRNA expression of *Hif1a* and its downstream genes. In general, HIF-1α was stabilized and significantly increased in protein level followed by upregulation of the downstream genes after CoCl_2_ administration. As a result of the negative feedback from the post translational protein modification, *Hif1a* was rather downregulated in mRNA level by CoCl_2_ administration (Ibuki et al., 2019). Accordingly, in ARPE19 cells, *Hif1a* was significantly downregulated by administration of CoCl_2_ although administration of lactoferrin did not affect *Hif1a* expression (**Figure 2A**). The downstream genes of HIFs, such as *Pdk1*, *Vegfa*, and *Glut1* were upregulated by CoCl_2_. These gene expressions were not changed by lactoferrin administration (**Figures 2B–D**). In contrast, *Pdk1*, *Vegfa*, and *Glut1* (**Figures 3B–D**) were downregulated significantly by lactoferrin administration in 661W cells. Lactoferrin did not affect HIF-1α protein expression increased by CoCl_2_ administration in ARPE19 cells (**Figures 4A** and **S1A**, **B**) and 661W cells (**Figures 4B** and **S2A**, **B**). These data suggested that HIF signaling is significantly suppressed by lactoferrin beyond protein expression in retinal neuronal cells.

### Oral Administration of Lactoferrin Suppressed CNV Volume in the Laser CNV Model Mice

**Figure 5:**
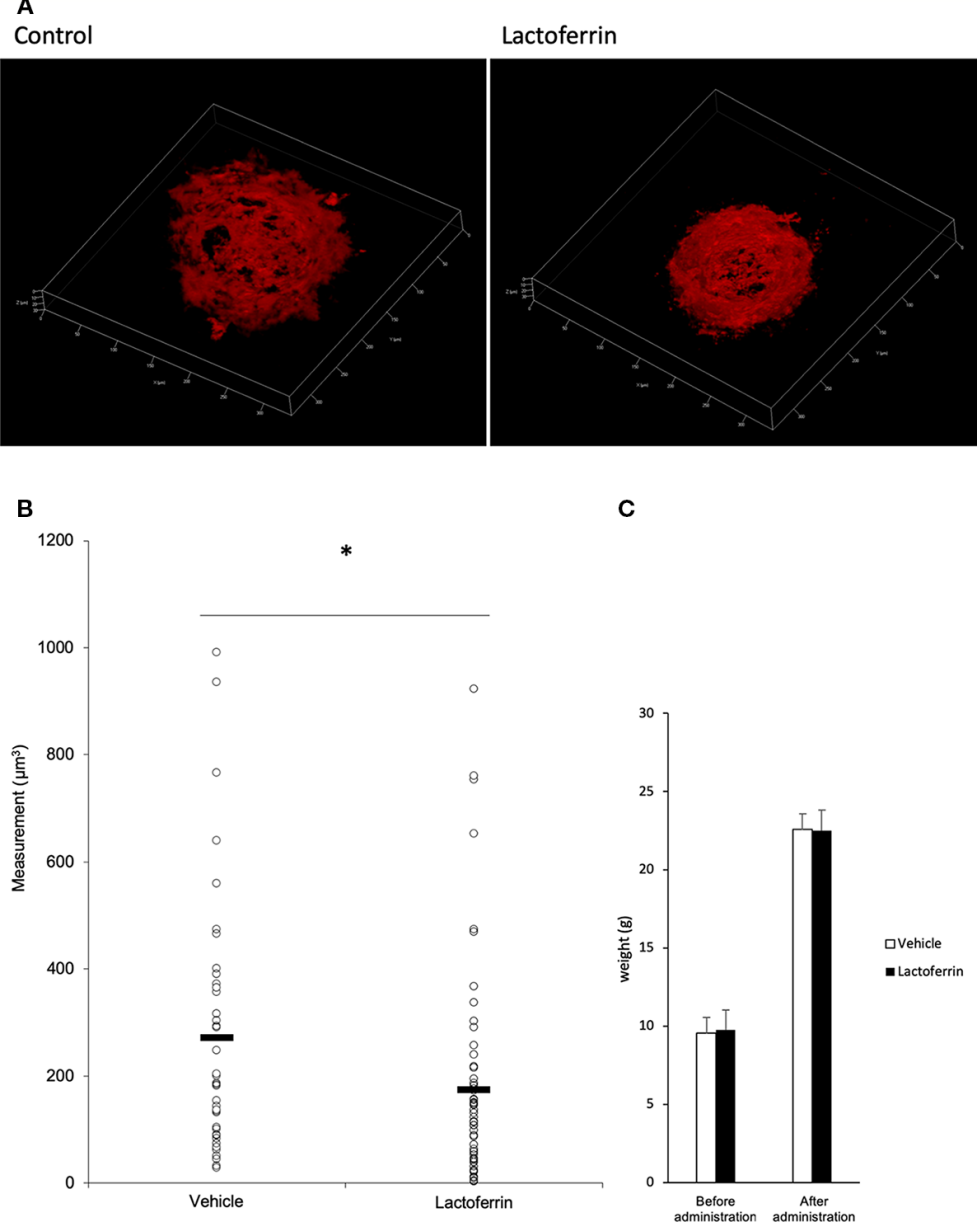
Oral administration of lactoferrin suppressed laser-induced choroidal neovascularization (CNV) volume in mice. **(A)** Representative three-dimensional images of the CNV stained with isolectin B4 (IB4). **(B)** Quantification of the CNV volume. Note that the administration of lactoferrin significantly reduced the CNV volume compared with the vehicle group. Vehicle: 272.731 ± 239.573 μm^3^, lactoferrin: 175.2 ± 196.13 μm^3^, six mice for each, *p < 0.05. **(C)** The average of the body weight before and after administration. Note that there was no significant change between the two groups. n = 6.

Lactoferrin was administered to the mice 6 days/week for a total of 5 weeks. We administered PBS for the vehicle group. We irradiated a laser 4 weeks after the beginning of the administration and evaluated the CNV volume on the seventh day after irradiation. A significant reduction in the CNV volume was observed in the lactoferrin group compared with the vehicle group (**Figures 5A, B**). We measured the body weight of the mice before and after administration, and there is no significant change observed between the vehicle- and lactoferrin-administrated groups (**Figure 5C**).

### Administration of Lactoferrin Suppressed HIF-1α Expression *In Vivo*

**Figure 6:**
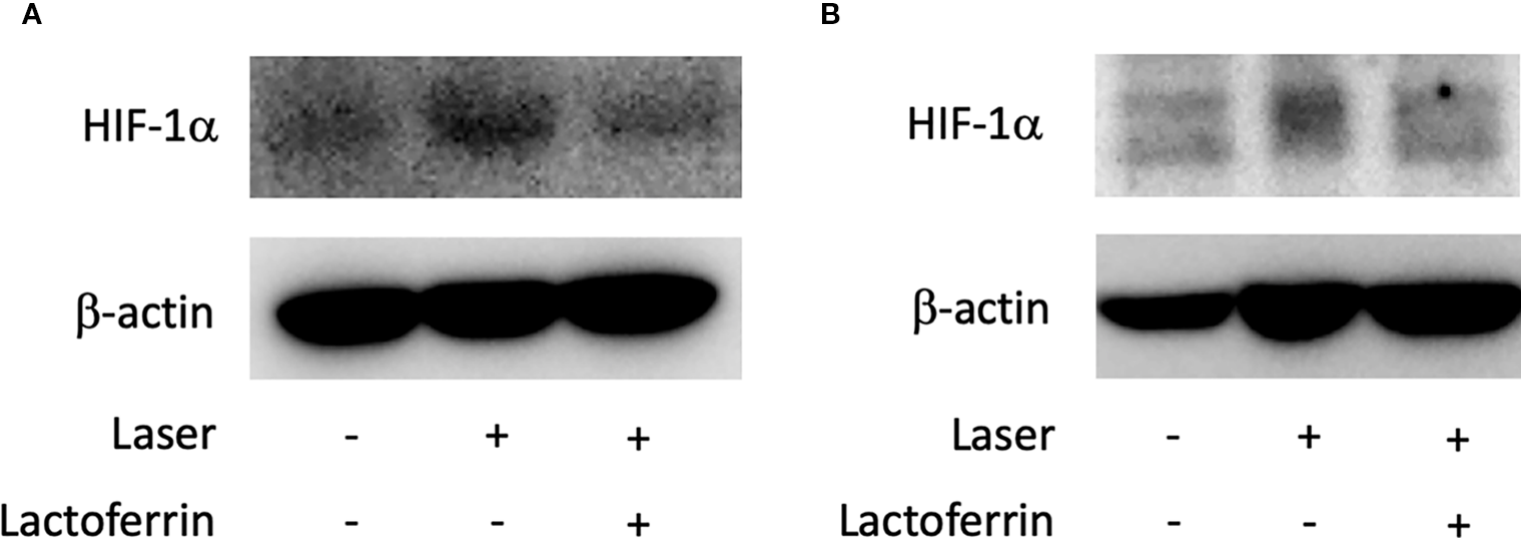
HIF-1α protein expression is suppressed by lactoferrin administration *in vivo*. Western blot for HIF-1α with the tissue samples from the retinal pigment epithelium (RPE)/choroid **(A)** and the retina **(B)**. Note that administration of lactoferrin suppressed HIF-1α expression, which increased with the laser irradiation in both the retina and RPE/choroid.

Lactoferrin dissolved in PBS was orally administered to the mice for a total of 31 days, and the mice were irradiated with a laser on the fourth week of administration. In the choroid (**Figures 6A** and **S3A**, **B**) and the retina (**Figures 6B** and **S3C**, **D**), HIF-1α protein was increased with the laser irradiation and suppressed by the administration of lactoferrin, even though the signal with the RPE/choroid tissue was weak (**Figure 6A**).

### CNV Volume Was Reduced in Both RPE and Neural Retina Specific *Hif1a* Conditional Knockout Mice

**Figure 7:**
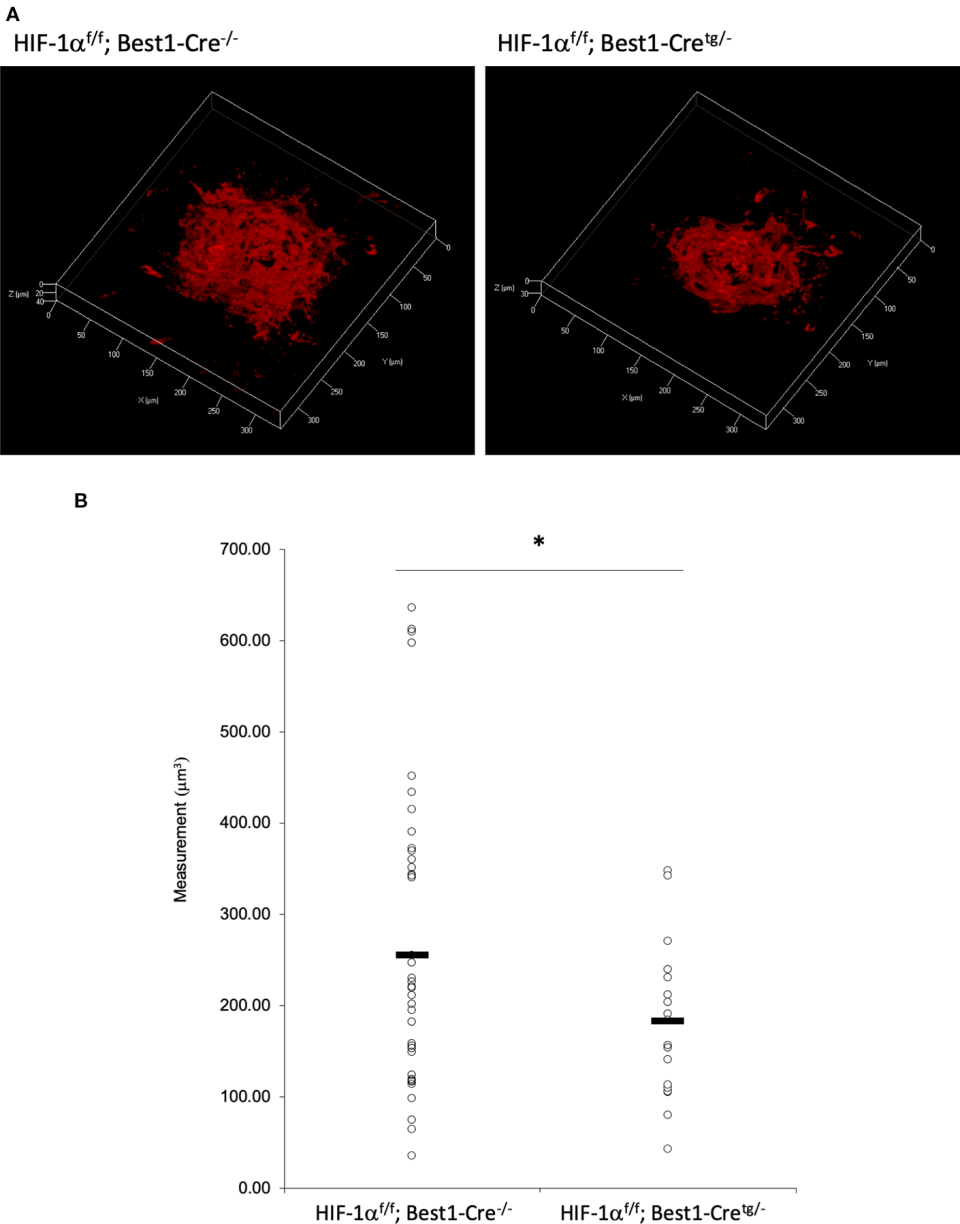
A significant reduction of the CNV volume in RPE specific *Hif1a* conditional knockout mice. **(A)** Representative three-dimensional images of the CNV stained by IB4. **(B)** Quantification of the CNV volume. Note that the CNV volume in *Hif1a* ^f/f^; Best1-Cre^tg/-^ mice was significantly reduced compared with the control. *Hif1a* ^f/f^; Best1-Cre^-/-^: 256.14 ± 160.37 μm^3^, *Hif1a* ^f/f^; Best1-Cre^tg/-^: 183.89 ± 91.26 μm^3^. Six mice for *Hif1a* ^f/f^; Best1-Cre^-/-^ and *Hif1a* ^f/f^; Best1-Cre^tg/-^, respectively. *p < 0.05. n = 6.

**Figure 8:**
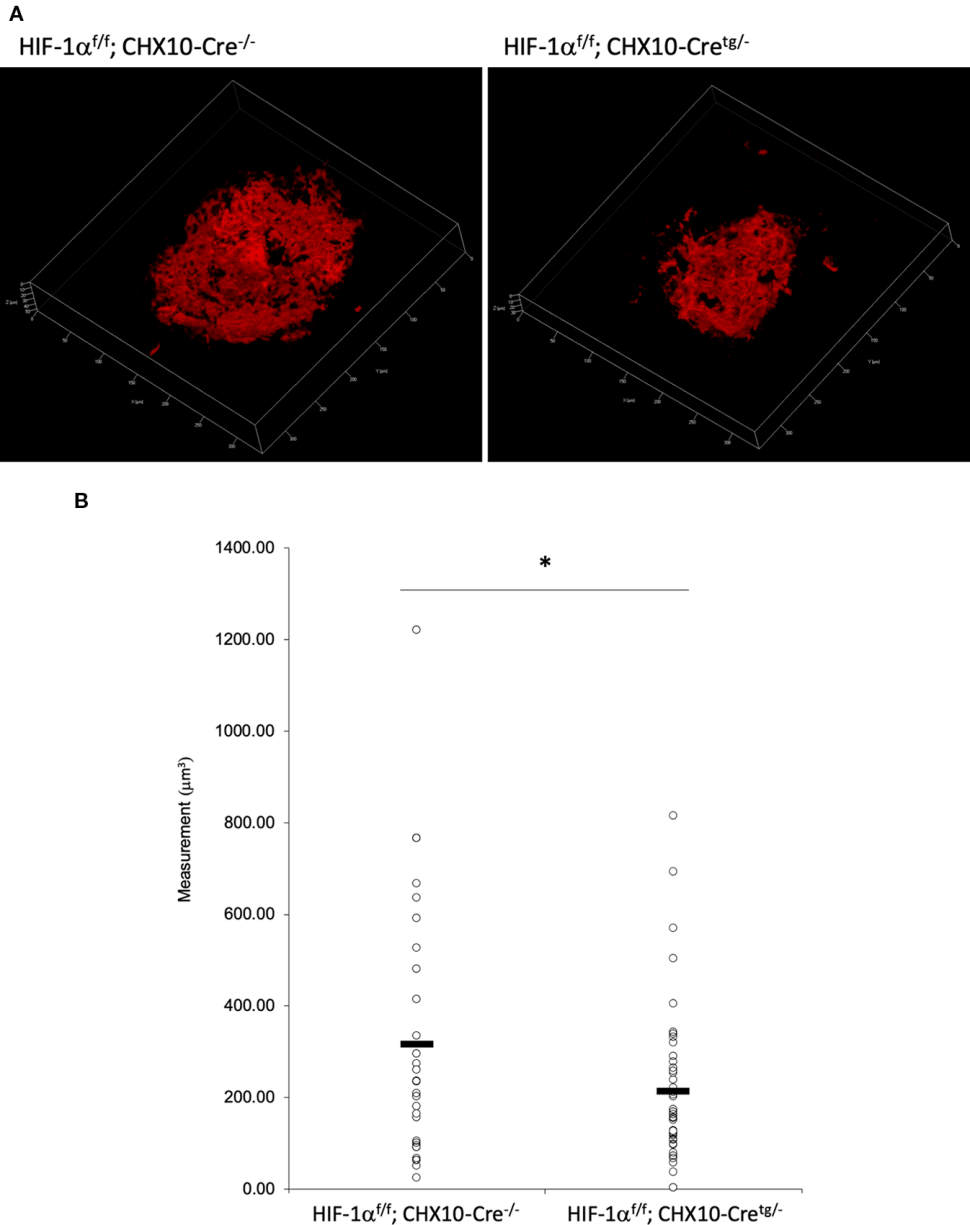
A significant reduction of the CNV volume in neural retina specific *Hif1a* conditional knockout mice. **(A)** Representative three-dimensional images of the CNV stained by IB4. **(B)** Quantification of the CNV volume. Note that the CNV volume in *Hif1a* ^f/f^; CHX10-Cre^tg/-^ mice was significantly reduced compared with the control. *Hif1a* ^f/f^; CHX10-Cre^-/-^: 317.84 ± 282.97 μm^3^, *Hif-1a* ^f/f^; CHX10-Cre^tg/-^: 214.74 ± 164.16 μm^3^. Six and four mice for *Hif1a* ^f/f^; CHX10-Cre^-/-^ and *Hif1a* ^f/f^; CHX10-Cre^tg/-^, respectively. *p < 0.05. n = 4–6.

To verify whether the CNV volume is regulated by HIF-1α expression, we examined the pathological phenotype in tissue specific *Hif1a* conditional knockout model mice. To target RPE cells or retinal neuronal cells specifically, we generated *Hif1a* ^f/f^; Best1-Cre mice and *Hif1a* ^f/f^; Chx10-Cre mice, respectively. As same as the previous report by utilizing VMD2-Cre mice (Kurihara et al., 2012), RPE specific *Hif1a* knockout mice showed a significant reduction of the CNV volume even with a different Cre transgenic mice line (**Figures 7A, B**). We also generated and examined sensory retina specific *Hif1a* knockout mice, showing a significant CNV reduction (**Figures 8A, B**). These data suggested that not only in RPE cells, but also in retinal neuronal cells, HIF-1α expression significantly affects CNV formation.

## Discussion

In this study, we revealed that lactoferrin has an HIF inhibitory effect in the 661W cone photoreceptor cell line, suppressing HIF activity and the downstream genes (**Figures 1** and **3**). The results of the luciferase assay indicated that lactoferrin potentially suppresses HIF activity in RPE cells as well; however, the suppressive effect was limited according to the downstream evaluation (**Figure 2**), indicating that this suppressive effect may be actuated in a cell type dependent manner.

Lactoferrin is an iron-binding protein which is a monomeric, 80-kDa glycoprotein, with a single polypeptide chain of about 690 amino acid residues. Its amino acid sequence relationships place it in the wider transferrin family (Baker and Baker, 2005). It is known that lactoferrin regulates the quantity of iron absorbed in the intestine *via* its role in iron transport, and it can chelate iron, directly or indirectly (Hao et al., 2019). Lactoferrin has been used as an adjuvant therapy for some intestinal diseases and is now used in nutraceutical supplemented infant formula and other food products (Hao et al., 2019). Lactoferrin also has other numerous biological roles, such as the modulation of immune responses and anti-microbial, anti-viral, antioxidant, anti-cancer, and anti-inflammatory activities (Hao et al., 2019).

Lactoferrin is also known to bridge innate and adaptive immune functions in mammals. It is a pleiotropic molecule that directly assists in the influence of presenting cells for the development of T-helper cell polarization (Actor et al., 2009). It has been reported that lactoferrin reduces oxidative stress-induced apoptosis (Actor et al., 2009), and that *Streptococcus mutans* and *Vibrio cholerae*, but not *Escherichia coli*, were killed by incubation with purified human apolactoferrin (Arnold et al., 1977). It has also been reported that lactoferrin injection inhibits staphylococcal kidney infections (Bhimani et al., 1999). Lactoferrin at high concentrations has an ability to promote growth and differentiation of the immature gut by enhancing proliferation of enterocytes and closure of enteric gap junctions, while at lower concentrations lactoferrin stimulates differentiation of enterocytes and expression of intestinal digestive enzymes (Buccigrossi et al., 2007). It has been reported that lactoferrin activates intestinal mucosal immunity in tumor-bearing mice (Wang et al., 2000). In addition, several *in vitro* studies have shown that lactoferrin is able to stimulate the growth of bifidobacteria; however, this effect is differentially exerted on different species and strains of bifidobacteria (Petschow et al., 1999; Liepke et al., 2002; Kim et al., 2004). In terms of anti-cancer effects, oral administration of bovine lactoferrin inhibits carcinogenesis in the colon and other organs in rats, and lung metastasis in mice (Iigo et al., 2009).

As described above, lactoferrin is known to have various roles. It has been reported that selenium-binding lactoferrin (Se-lactoferrin) eye drops suppress the upregulated expression of heme oxygenase-1, cyclooxygenase-2, matrix metallopeptidase-9, and interleukin-6, and also suppress 8-OHdG production in a murine dry eye model induced by surgical removal of the lacrimal glands (Higuchi et al., 2012). Se-lactoferrin eye drops have also been shown to have efficacy in a tobacco smoke exposure-induced rat dry eye model and a short-term rabbit dry eye model (Higuchi et al., 2016). It is also reported that oral lactoferrin administration preserves lacrimal gland function in aged mice by attenuating oxidative damage and suppressing subsequent gland inflammation (Kawashima et al., 2012). In the current study, we found that oral administration of lactoferrin has a therapeutic effect in a laser-induced CNV model mimicking the neovascular type of AMD (**Figure 5**). This result is consistent with the previous report that the CNV volume is significantly increased in *lactoferrin* gene knockout mice (Montezuma et al., 2015). It has been reported that HIF downstream genes were upregulated in a laser-irradiated RPE/choroid (Kurihara et al., 2012). Oral administration of lactoferrin suppresses the increased HIF-1α expression both in the RPE and the neural retina (**Figure 6**). Thus, there may be some dissociation of lactoferrin action against HIF-1α between *in vitro* and *in vivo* observations in the current experiments. We speculate that this is because lactoferrin may be metabolized in the body to directly suppress HIF-1α protein expression in the eye. We further confirmed that not only in the RPE (**Figure 7**), but also in the neural retina (**Figure 8**), HIF-1α expression significantly contributes to CNV formation. These results indicate that lactoferrin suppresses CNV formation by suppressing HIF-1α in PRE and the neural retina.

In conclusion, HIF-1α inactivation either in RPE or the neural retina can suppress CNV formation in a murine laser irradiation model. CNV formation is suppressed by oral administration of lactoferrin *via* HIF-1α inactivation in the RPE and neural retina. These results suggest a potential clinical use of lactoferrin in daily life to prevent AMD.

## Data Availability Statement

All datasets generated for this study are included in the article/**Supplementary Material**.

## Ethics Statement

The animal study was reviewed and approved by the Institutional Animal Care and Use Committee at Keio University.

## Author Contributions

MI performed all the experiments. CS and YM established the experimental protocols. AI prepared the experimental materials. MI and TK contributed to the conception and design of the study. TK and KT supervised the project. All authors approved the final version for submission.

## Funding

This work was funded by Grants-in-Aid for Scientific Research (KAKENHI, number 15K10881 and 18K09424) from the Ministry of Education, Culture, Sports, Science and Technology (MEXT) to TK. This study was conducted with financial support from ROHTO Pharmaceutical. The authors declare that this study received funding from ROHTO Pharmaceutical (Osaka, Japan). The funder was not involved in the study design, collection, analysis, interpretation of data, the writing of this article or the decision to submit it for publication.

## Conflict of Interest

Patents have been applied for field relating to the therapeutic effects of lactoferrin in ocular disorders. KT holds the position of CEO of Tsubota Laboratory, Inc.

The remaining authors declare that the research was conducted in the absence of any commercial or financial relationships that could be construed as a potential conflict of interest.
