# Supplementary figures and images for: Lactoferrin Has a Therapeutic Effect via HIF Inhibition in a Murine Model of Choroidal Neovascularization
Source: Front Pharmacol. 2020 Feb 28;11:174. doi: 10.3389/fphar.2020.00174 (PMC7059857; doi:10.3389/fphar.2020.00174)

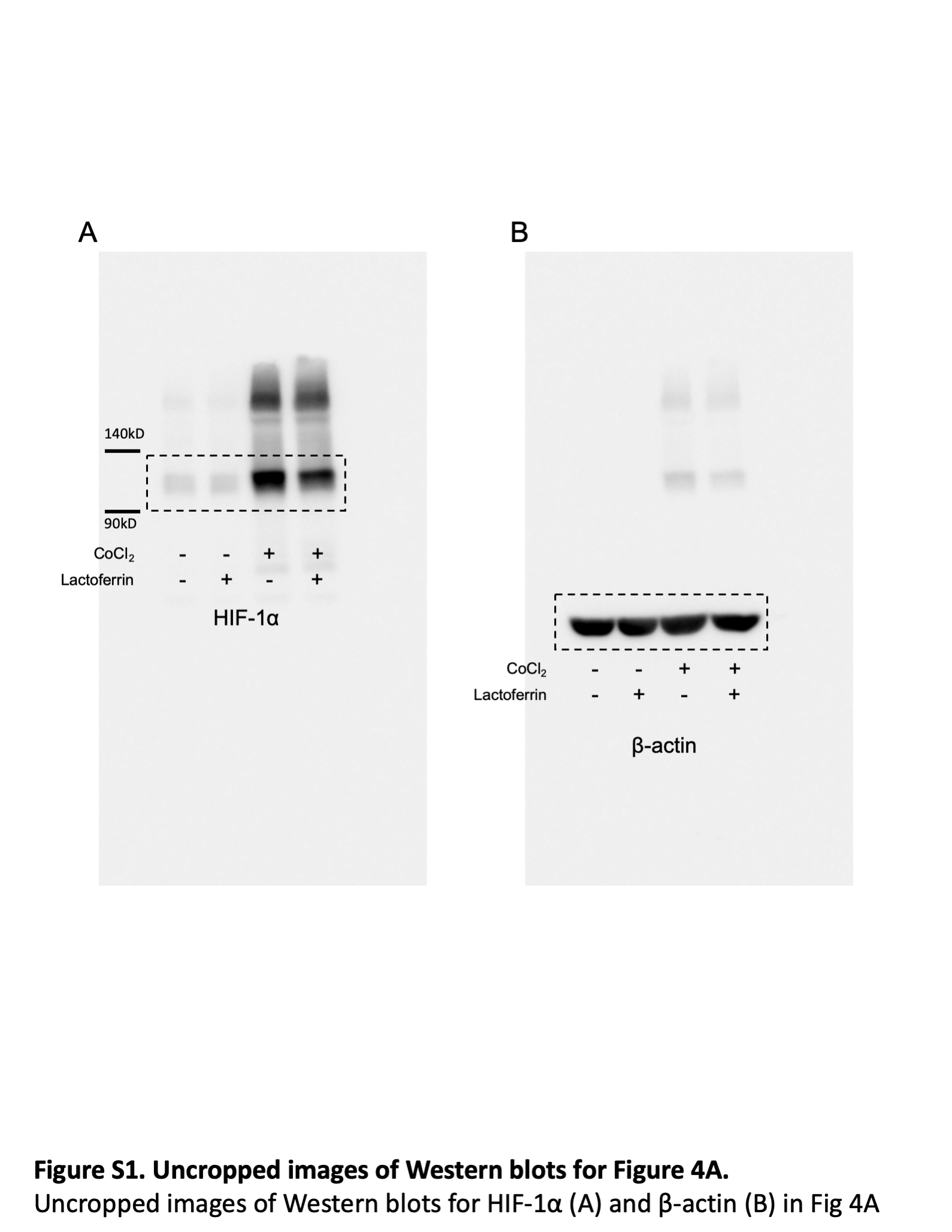

Supplement: Supplementary file 1 [file Image_1.tiff]

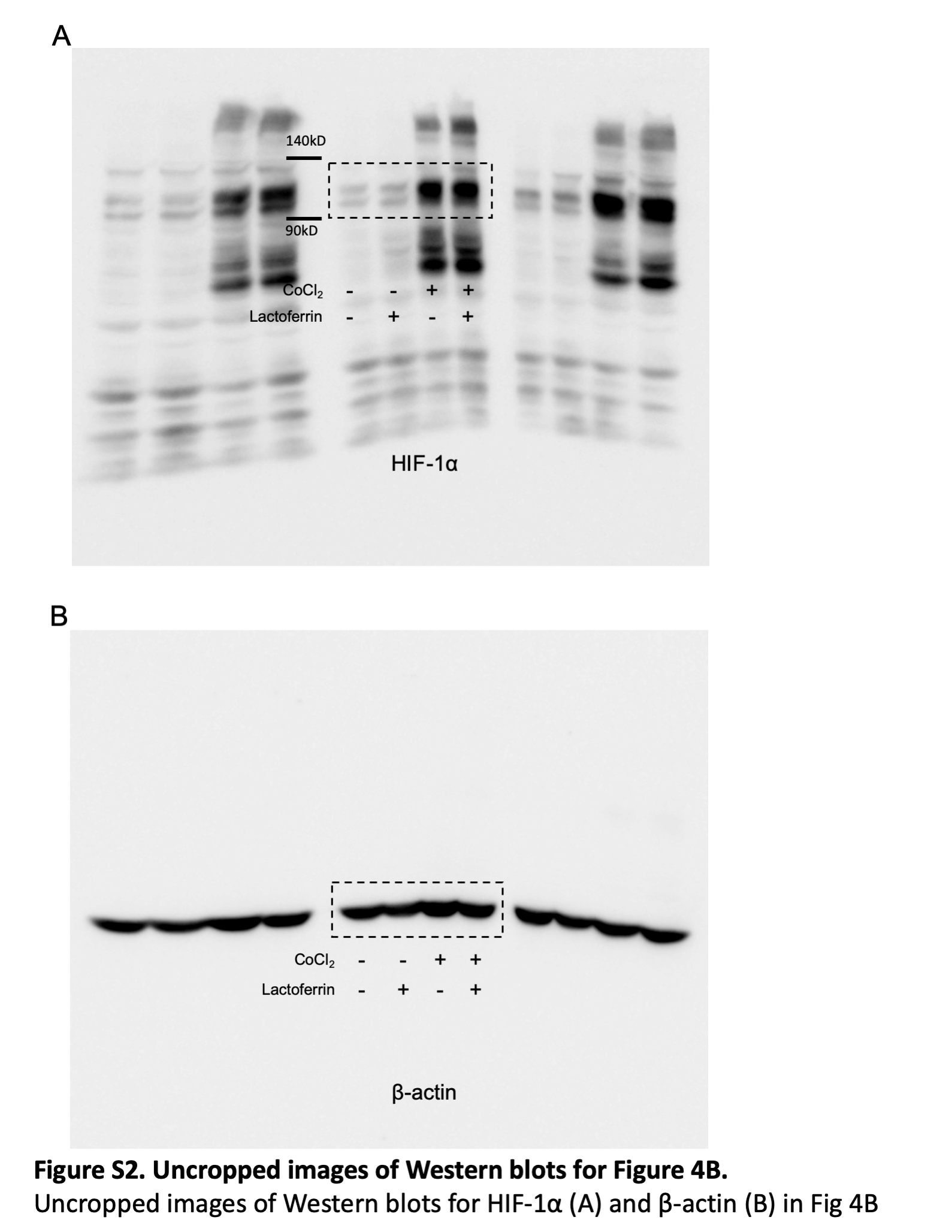

Supplement: Supplementary file 2 [file Image_2.tiff]

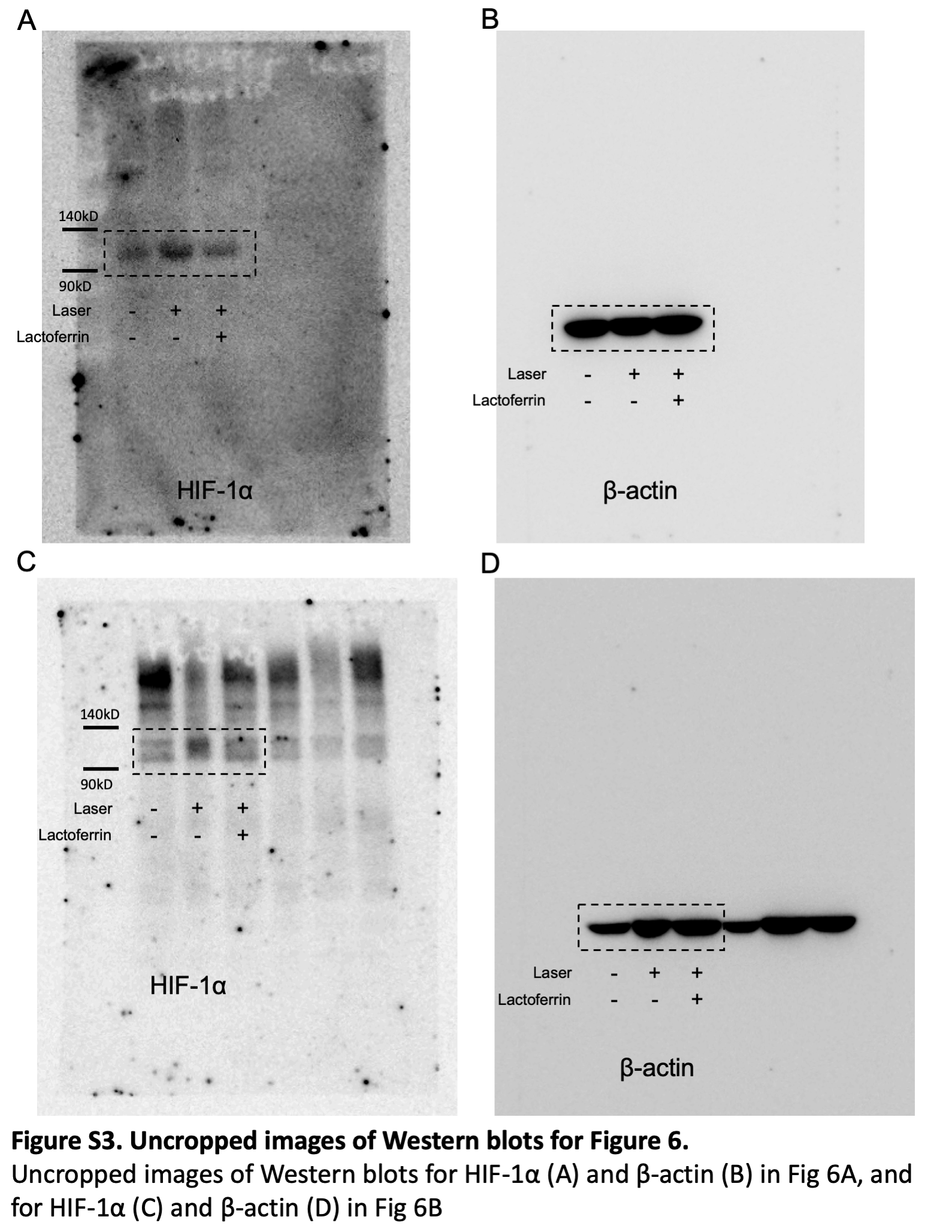

Supplement: Supplementary file 3 [file Image_3.tiff]

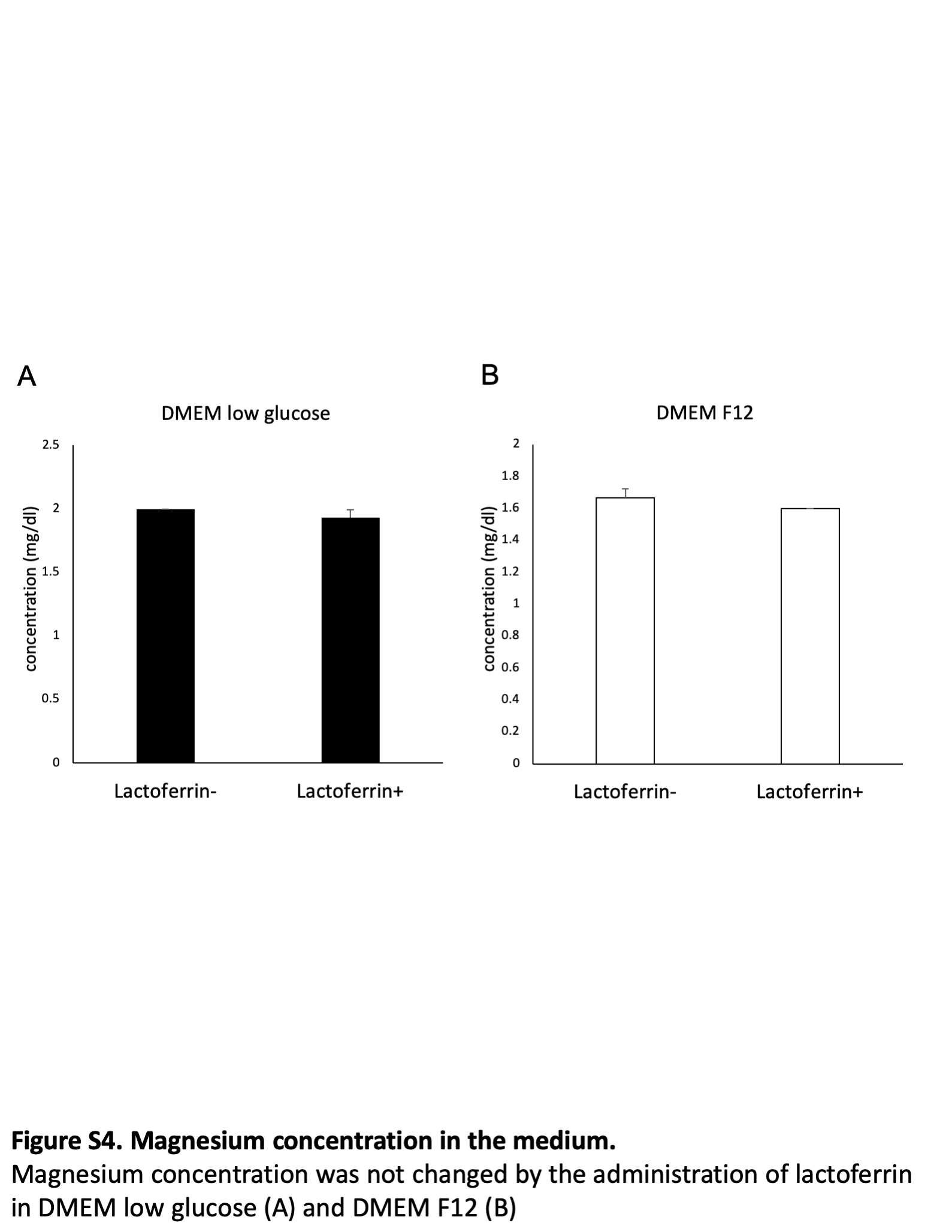

Supplement: Supplementary file 4 [file Image_4.tiff]
